# Listening loops and the adapting auditory brain

**DOI:** 10.3389/fnins.2023.1081295

**Published:** 2023-03-16

**Authors:** David McAlpine, Livia de Hoz

**Affiliations:** ^1^Department of Linguistics, Macquarie University, Sydney, NSW, Australia; ^2^Neuroscience Research Center, Charité – Universitätsmedizin Berlin, Berlin, Germany; ^3^Bernstein Center for Computational Neuroscience, Berlin, Germany

**Keywords:** auditory, listen, loops, feedback, adaptation, prediction

## Abstract

Analysing complex auditory scenes depends in part on learning the long-term statistical structure of sounds comprising those scenes. One way in which the listening brain achieves this is by analysing the statistical structure of acoustic environments over multiple time courses and separating background from foreground sounds. A critical component of this statistical learning in the auditory brain is the interplay between feedforward and feedback pathways—“listening loops”—connecting the inner ear to higher cortical regions and back. These loops are likely important in setting and adjusting the different cadences over which learned listening occurs through adaptive processes that tailor neural responses to sound environments that unfold over seconds, days, development, and the life-course. Here, we posit that exploring listening loops at different scales of investigation—from *in vivo* recording to human assessment—their role in detecting different timescales of regularity, and the consequences this has for background detection, will reveal the fundamental processes that transform hearing into the essential task of listening.

## The act of listening

Our brain is continuously interpreting the soundscape, it is listening even when we are not. Listening is essential to understanding. Without listening, sound is meaningless to us—a wash of noise, reflections, and competing sources vying for our attention. Many of our listening environments are challenging—from restaurants to railway stations, we listen in complex, multi-sensory and multi-dimensional spaces. Compared to even the most advanced listening technologies, however, we navigate these spaces with relative ease, and it is not obvious how we do so. We evolved to deal with listening in an embodied manner but our experimental approaches, and often our listening technologies, pay little regard to the immersive and embodied qualities of listening. A reductionist approach to our exploration of the listening brain will limit the development of algorithms, devices, and therapies that seek to establish or re-establish listening—in humans and machines—as an immersive experience.

**FIGURE 1:**
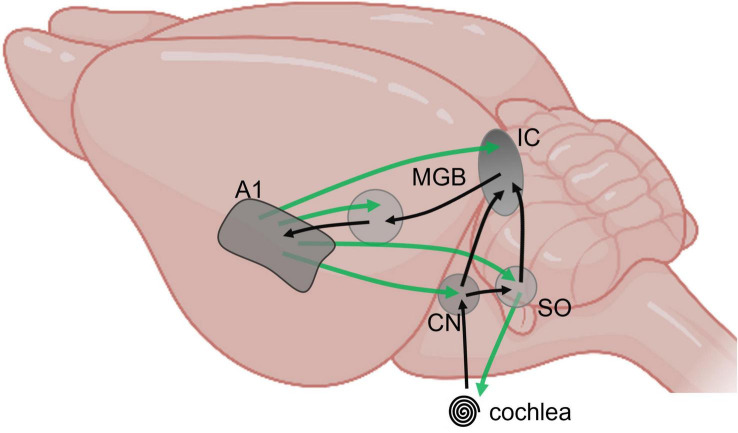
Schematic representation of major feedforward (black) and feedback (green) pathways between subcortical and cortical auditory structures, from cochlea to primary cortex, projected on a mouse brain (BioRender).

Here, we posit that advancing our understanding of the listening brain requires a reframing of our investigative neuroscience to include both the multi-layered soundscape with its noisy background as well as its complex foreground. In doing so, we will have to contend with the complexities of an extensive neural circuit and the specific features of the auditory pathway—evident from cochlea to cortex and back—the “listening loops” responsible for setting the cadences of our listening lives (Winer, 2005; Asilador and Llano, 2020). Sensitivity to salient foreground acoustic cues is important for processing speech information, for example, but background features such as multi-talker babble or the flurry of late-arriving reflections from walls and other surfaces in a room also need to be integrated into our listening experience. Exploring how the listening brain parses background features of the soundscape is critical to survival—fight or flight—since this sensitivity to the statistical structure of background sounds may also enhance our capacity to attend to foreground sounds. Here we posit that studying the mechanisms underlying the detection and coding of the background is essential to understand listening (McWalter and McDermott, 2018). What are the statistics of the background that facilitate its detection? How is it coded? What is the role of feedback? And on which time scale? How and when does its coding depend on contextual information (spatial context, movement, visual stimuli)? Learning the longer-term statistical structure of acoustic environments involves an interplay between feedforward and feedback pathways—the listening loops—including to the level of the inner ear, which takes us directly to the issue of how to explore listening through, and in the context of the complex neural circuits that constitutes the auditory brain. Though afferent, or feedforward, pathways in the auditory brain are rightly considered vital at the juncture between hearing and cognition, feedback (efferent) fibres outnumber feedforward in the auditory brain to influence every station in the pathway, including mechanical and neural structures within the middle and inner ear (Saldaña et al., 1996; Terreros and Delano, 2015). The functional understanding of these cortico-subcortical loops lags well behind our knowledge of their anatomy. Overall, it seems reasonable to assume that the act of listening arises from activity generated in a rich subcortical network replete with bilateral and feedback connectivity, and that this activity operates over progressively wider time windows along the ascending pathway (Ding et al., 2016; Kell and McDermott, 2019; Asokan et al., 2021; Henin et al., 2021), with feedback from relatively higher centres in the auditory pathway modulating neural activity at lower centres over potentially progressively longer epochs (Robinson et al., 2016; Figure 1). Understanding the functional role of cortico-subcortical listening loops in the human brain could support the many autonomous listening devices—from hearing aids and cochlear implants to Amazon’s “Alexa”—that currently provide little of the capacity of human listening abilities. Striving for signal fidelity on millisecond, and even sub-millisecond, timescales, they often struggle to perform in even moderately noisy environments, and fail to operate over the multiple, and much slower, cadences of listening that make effective communication possible. The dominance of rapid signal-processing techniques in the development of hearing technologies and therapies, also surfaces in machine-learning and artificial intelligence approaches to listening. Performance remains distinctly *subpar* but progress on this front will be critical if autonomous listening devices.

## Tools for exploring listening loops

If we are to take advantage of listening loops to explore the timescales over which sensory information is integrated in the auditory brain (Ding et al., 2016) we may need to implement some new tools to do so. One difficulty in studying cortico-subcortical loops has been the sampling and targeting of, not only deep-sitting neurons, but also those specifically involved in the loop. Thanks to the development of genetic tools, combined with the creation of manipulation tools, we can now opto- and chemo-genetically target deep and superficial cells specifically involved in cortico-subcortical interactions in awake rodents (Clayton et al., 2021; Souffi et al., 2021). The use of the newly developed large-scale high-density recording probes (Jun et al., 2017) allows to record activity from deep and superficial neurons simultaneously across structures (Kleinfeld et al., 2019). The advent of brain-imaging techniques, with the potential to sample from wide populations of neurons within and across brain structures (Bathellier et al., 2012; Silva, 2017), has put hearing on a more equal footing to other sensory systems, particularly vision, for which an understanding of cortical structure and function was well advanced through *in vivo* experimentation (Hübener and Bonhoeffer, 2005). Imaging of the auditory brain has rapidly advanced from employing simple sounds that build on our understanding of sensory reception and the importance of spectral analysis—tonotopy is widely accepted as the primary representation of the cochlea (Marin et al., 2022)—to more-naturalistic listening assessments permitted by advances in audio technologies (Filipchuk et al., 2022). The downside of current brain-imaging techniques, however, is that they still favour a cortico-centric perspective, with some exceptions (Barnstedt et al., 2015), at a time when subcortical structures and efferent pathways are increasingly understood to be critical to the act of listening (Cruces-Solís et al., 2018). In *in vivo* experimental settings, two-photon imaging is generally confined to the exploration of cortical structures—though this is changing with the implementation of mesoscale imaging techniques. However, access to subcortical structures—some deep within the brainstem—as well investigations of the efferent pathways, remain limited, especially in humans. Further, many practical limitations of imaging arise beyond the inability to access subcortical structures. The (dangerously loud) sounds generated by magnetic resonance imaging (MRI) scanners pose a specific challenge to structural and functional investigations of the listening brain *per se*, but MRI as well as magnetoencephalography (MEG) are contra-indicated for the use of the very listening devices that might provide powerful insights to hearing and listening in health and disease.

## Listening loops and the adapting brain

Exploring the auditory brain in terms of listening loops conditioned for effective sensing and communication with the outside world is, in fact, how the research field is starting to align (Bajo et al., 2010; Robinson et al., 2016; Weible et al., 2020; Yudintsev et al., 2021; Wang et al., 2022), powered by a combination of new technologies applied generally across sensory neuroscience (e.g. Zingg et al., 2017; Williamson and Polley, 2019), and a specific re-imagining of the structure and function of subcortical auditory structures (Xiong et al., 2015; Bidelman et al., 2018; Lohse et al., 2021). Freed from a cortico-centric approach, the concept of listening loops provides the time-dimensional perspective to understanding, or at least exploring, the different cadences of listening (Antunes and Malmierca, 2021; Homma and Bajo, 2021), and connects with the well-developed concept of the predictive brain. Indeed, despite the technical challenges of accessing sub-cortical structures, the concept of listening loops that operate over distinct feedforward and feedback pathways provides an excellent framework in which to investigate fundamental principles of brain processing such as predictive coding that might be applied to other sensory systems, not generally a role the auditory system has performed.

One means by which the temporal dynamics of the listening brain might be investigated, including its capacity for prediction, is by assessing how it adapts over time to enhance the flow of information (Latimer et al., 2019). We can define adaptation to mean changes (usually a reduction) in neural firing in response to sustained stimulation, though definitions of the term are plentiful. From a functional perspective, firing-rate adaptation seems important in the listening brain’s ability to adjust dynamically to the listening environments in response to changes in that environment, or in response to internal changes that alter its overall sensitivity or dynamics. Adaptive coding is a common phenomenon throughout the brain, and a recent review article provides an excellent primer for understanding the different cadences over which adaptation in the auditory brain unfolds, from the range of milliseconds to over the life-course, as well as potential mechanisms by which these cadences are set or arise (Willmore and King, 2022).

Continuous adaptation within listening loops likely sets and adjusts the cadences over which learned listening occurs, tailoring neural responses to sound environments that unfold over seconds, days, development, and the life-course. Exploring these loops in the context of the adapting auditory brain—from single neurons in animal models to human behavioural assessments—will help us understand the immersive quality of listening, as well as advance the many technologies currently available or under development that purport to listen to us.

## Data availability statement

The original contributions presented in this study are included in the article/supplementary material, further inquiries can be directed to the corresponding author.

## Author contributions

Both authors conceptualised, wrote, and edited the manuscript.
