# Corrigendum: Listening loops and the adapting auditory brain

**DOI:** 10.3389/fnins.2023.1228450

**Published:** 2023-06-19

**Authors:** David McAlpine, Livia de Hoz

**Affiliations:** ^1^Department of Linguistics, Macquarie University, Sydney, NSW, Australia; ^2^Neuroscience Research Center, Charité – Universitätsmedizin Berlin, Berlin, Germany; ^3^Bernstein Center for Computational Neuroscience, Berlin, Germany

**Keywords:** auditory, listen, loops, feedback, adaptation, prediction

In the published article, there was an error regarding the affiliation for David McAlpine. As well as having affiliation 1, he should also have affiliation 2: Neuroscience Research Center, Charité – Universitätsmedizin Berlin, Berlin, Germany.

The authors apologize for this error and state that this does not change the scientific conclusions of the article in any way. The original article has been updated.

